# Asking about Sex in General Health Surveys: Comparing the Methods and Findings of the 2010 Health Survey for England with Those of the Third National Survey of Sexual Attitudes and Lifestyles

**DOI:** 10.1371/journal.pone.0135203

**Published:** 2015-08-07

**Authors:** Philip Prah, Anne M. Johnson, Anthony Nardone, Soazig Clifton, Jennifer S. Mindell, Andrew J. Copas, Chloe Robinson, Rachel Craig, Sarah C. Woodhall, Wendy Macdowall, Elizabeth Fuller, Bob Erens, Pam Sonnenberg, Kaye Wellings, Catherine H. Mercer

**Affiliations:** 1 Research Department of Infection and Population Health, University College London, London, United Kingdom; 2 Public Health England, London, United Kingdom; 3 NatCen Social Research, London, United Kingdom; 4 Department of Epidemiology and Public Health, University College London, London, United Kingdom; 5 Department of Social and Environmental Health Research, London School of Hygiene and Tropical Medicine, London, United Kingdom; 6 Department of Health Services Research and Policy, London School of Hygiene & Tropical Medicine, London, United Kingdom; Leibniz Institute for Prevention Research and Epidemiology (BIPS), GERMANY

## Abstract

**Objectives:**

Including questions about sexual health in the annual Health Survey for England (HSE) provides opportunities for regular measurement of key public health indicators, augmenting Britain's decennial National Survey of Sexual Attitudes and Lifestyles (Natsal). However, contextual and methodological differences may limit comparability of the findings. We examine the extent of these differences between HSE 2010 and Natsal-3 and investigate their impact on parameter estimates.

**Methods:**

Complex survey analyses of data from men and women in the 2010 HSE (n = 2,782 men and 3,588 women) and Natsal-3 undertaken 2010–2012 (n = 4,882 men and 6,869 women) aged 16-69y and resident in England, both using probability sampling, compared their characteristics, the amount of non-response to, and estimates from, sexual health questions. Both surveys used self-completion for the sexual behaviour questions but this was via computer-assisted self-interview (CASI) in Natsal-3 and a pen-and-paper questionnaire in HSE 2010.

**Results:**

The surveys achieved similar response rates, both around 60%, and demographic profiles largely consistent with the census, although HSE participants tended to be less educated, and reported worse general health, than Natsal-3 participants. Item non-response to the sexual health questions was typically higher in HSE 2010 (range: 9–18%) relative to Natsal-3 (all <5%). Prevalence estimates for sexual risk behaviours and STI-related indicators were generally slightly lower in HSE 2010 than Natsal-3.

**Conclusions:**

While a relatively high response to sexual health questions in HSE 2010 demonstrates the feasibility of asking such questions in a general health survey, differences with Natsal-3 do exist. These are likely due to the HSE’s context as a general health survey and methodological limitations such as its current use of pen-and-paper questionnaires. Methodological developments to the HSE should be considered so that its data can be interpreted in combination with those from dedicated sexual health surveys, thus improving our ability to monitor trends in sexual health.

## Introduction

Repeated population estimates of key sexual behaviours are necessary for informing and evaluating sexual health policy and practice.[1] Data are required to understand changing lifestyles, to monitor the impact of uptake of services and interventions, and to modify policy appropriate to the population’s attitudes and lifestyles. Probability sample surveys of the general population provide the most reliable population prevalence estimates in this context,[1–4] and are preferable to using convenience sampling of often unrepresentative online panels [5] or high-risk groups recruited from specific venues [6] or health services,[4] but the frequency of such surveys is limited by financial costs.[1–4]

The National Surveys of Sexual Attitudes and Lifestyles (Natsal), probability sample surveys of men and women in Britain conducted decennially since 1990, collect detailed data on sexual behaviours and have been used extensively to inform public health policy and practice.[7,8] Until 2010, Natsal was the only source of population estimates for such behaviours in the Britain. The third Natsal, carried out between 2010 and 2012, identified and quantified trends in risk behaviour, risk reduction practices and adverse sexual health outcomes.[9,10] However, estimates from a survey undertaken relatively infrequently may quickly become out-of-date, and so adding a sexual health module to a regular general health survey, such as the Health Survey for England (HSE), may be beneficial for behavioural surveillance,[11] as well as enabling linking of these data to biological markers collected in HSE.

Begun in 1990, the HSE is an annual nationally-representative general health survey in which participants are asked questions relating to a range of factors including demographic and socio-economic data, health, social care and lifestyle risk factors. In 2010, for the first time, HSE included a module of questions relating to sexual health, many of which replicated those used in Natsal.

A number of differences exist between HSE and Natsal that might influence the comparability of prevalence estimates between the surveys. Differences can be categorised as contextual: as a general health survey those agreeing to take part in HSE may differ to those agreeing to participate in Natsal, and also methodological: for instance, HSE used a pen-and-paper self-completion for the sexual health module whilst Natsal used a computer-assisted-self-interview (CASI). Furthermore, the HSE surveys several adults within a household and can interview multiple people concurrently, whilst Natsal selects just one individual per household to interview. These differences may affect willingness to report in terms of item non-response as well as overall response,[11,12] but the extent to which this is the case is unknown. To address this, we compare HSE 2010 and Natsal-3 in terms of: i) participants’ sociodemographic and general health characteristics, and their similarity to census data; ii) the proportion of participants in each survey who did not answer the sexual health questions; and iii) the extent to which sexual health estimates differ.

## Methods

### Data

The HSE is an annual survey monitoring the health of the general population living in private households in England.[13] A new sample is selected each year. Details of the 2010 survey (HSE 2010) have been published elsewhere.[14] Briefly, fieldwork was conducted by Natcen Social Research between 1/2010 and 2/2011 and adopted a multi-stage, stratified probability sampling design, using addresses from the small user Postcode Address File (PAF) as the sampling frame. All adults (aged 16+) living in selected households were invited to participate in the survey. Data were weighted to the 2009 mid-year population estimates according to age, sex, region, household type and social class. Questionnaires were administered using a combination of face-to-face computer-assisted-personal-interview (CAPI) and a self-completion pen-and-paper-interview (PAPI) booklet with the interviewer present. The sexual health module was administered to all adults aged 16–69 years within the self-completion booklet, and participants had no prior knowledge of these questions. Booklets were sealed in an envelope upon completion and returned to the interviewer.

The National Survey of Sexual Attitudes and Lifestyles (Natsal) is a decennial survey, primarily intended to measure sexual behaviour and attitudes of men and women living in private households in Britain. Detailed methodology has been published elsewhere.[9,15] Fieldwork was conducted by Natcen Social Research between 9/2010 and 8/2012. The third Natsal (Natsal-3) sampled households from the same PAF as HSE 2010, but just one individual aged 16–74 was randomly selected from each household and invited to participate. Data were weighted to account for unequal probabilities of selection and for non-response to match the age, sex and region distributions according to the 2011 Census. Natsal-3 used both face-to-face CAPI and computer-assisted-self-interview (CASI), for the more sensitive questions with the interviewer present throughout.

Analyses here are restricted to participants aged 16–69 years and resident in England in both HSE 2010 and Natsal-3.

### Ethics statements

All Natsal-3 participants were given an information leaflet to read prior to participating in the survey and had the opportunity to discuss this with the interviewer. Verbal informed consent was obtained for participation in the interview and interviewers had to confirm that respondents had read the information leaflet in the computer programme before commencing the interview. The Natsal-3 study, including the consent procedures, was approved by the Oxfordshire Research Ethics Committee A (reference: 09/H0604/27). All participants provided their own consent to participate, however for 16–17 year olds living at home, a parent/guardian provided additional verbal assent for participation.

For HSE 2010, research ethics approval was obtained from Oxford B Research Ethics Committee (ref 09/H0605/73). All participants were given information in an advance letter and three information leaflets about the survey in general, the interviewer visit, and the nurse visit. All participants gave verbal consent to participate in the interview.

### Data availability

Due to the large amount of detailed data in the Natsal-3 dataset, including information from survey participants which is of a highly-sensitive nature, great care is needed when preparing a publically-available dataset in order to avoid potential breach of confidentiality. At the time of writing, final preparations are being made to archive the >1,600 variables in the Natsal-3 dataset in July 2015 at which point these data will be publicly-available from the UK Data Archive (www.data-archive.ac.uk). In the meantime, researchers can contact the Natsal team to request secure access to the Natsal-3 dataset, including those variables used for the analyses presented in this paper. The contact details can be found here: http://natsal.ac.uk/natsal-3/data-archiving.aspx.

The HSE 2010 data are already archived and publicly available from: http://discover.ukdataservice.ac.uk/catalogue/?sn=6986&type=datacatalogue.

### Measures

Data only for variables with similar question wording in the HSE 2010 and Natsal-3 were included in these analyses to permit reliable comparisons (S1 Table). Demographic characteristics included age, marital status, ethnicity, socio-economic class (defined using the five-category National Statistics Socioeconomic classification (NS-SEC)),[16] highest level of education, and household size. Health indicators included self-perceived general health status, longstanding illness or disability, frequency of binge drinking (more than six (women) or eight (men) units in one occasion), and smoking cigarettes. Sexual behaviour and STI related indicators included: age at first heterosexual sexual intercourse, number of sexual partners (lifetime and past year), same-sex experience (ever and past 5 years), ever paying for sex, testing for chlamydia in the past year and ever been diagnosed with an STI (excluding thrush). Contraception questions asked about usual use at present of the contraceptive pill, male condom, and male and female sterilisation. Demographic and health questions were asked during face-to-face section of the interview in both surveys. All sexual health questions in HSE 2010 were included in the pen-and-paper self-completion questionnaire, whilst they were included in the CASI in Natsal-3, with the exception of the questions relating to first sexual intercourse and contraception, which were asked face-to-face using showcards.

### Statistical methods

All analyses were performed using the complex survey analysis functions in Stata 13.1 (StataCorp LP, College Station, Texas) to take into account stratification, clustering, and weighting of the data in each survey.

We report the overall individual response rate for the two surveys, as well as the response to the self-completion modules. The demographic characteristics of the two survey samples were compared with the 2011 Census estimates (for England only), and to one another to examine differences in the characteristics of participants. This part of our analysis was restricted to participants aged 16–64 years as Census data for our complete age range is not available (all other analyses used the complete 16–69 years age range). Differences in item non-response were investigated by comparing the percentage of participants in each survey who were asked, but did not answer, the sexual health questions. We present prevalence estimates for the sexual health questions and used logistic regression to measure the extent to which response to these questions differs in HSE 2010 relative to Natsal-3. We then used multivariable models to adjust for significant demographic confounders between the surveys. We investigated age-group interactions to see if differences between the surveys varied by age. Additionally, we repeated the analysis comparing prevalence estimates for the sexual health questions after restricting our sample to those participants in the two surveys who lived alone to take account of the possible reporting bias in HSE 2010 from participating in the presence of other household members completing the survey at the same time, given the sensitive nature of the questions. All analyses were stratified by gender.

## Results

### Survey response

The response rates to HSE 2010 for all adults aged 16+ and Natsal-3 for adults aged 16–74 were similar, 59% and 58%, respectively.[14,15] A total of 6,370 adults (2,782 men and 3,588 women) aged 16–69 years participated in HSE 2010, and there were 11,751 participants (4,882 men and 6,869 women) aged 16–69 years and resident in England in Natsal-3. The analyses presented hereon are based on these participants.

### Participants’ demographic and health characteristics

Prior to non-response weighting, we found that participants recruited for HSE 2010 under-represented young men and women but over-represented older men and women (Tables 1 and 2). To a lesser extent this trend is replicated in Natsal-3: men and women aged 25–34 were under-represented whilst men aged 55–64 and women aged 35–54 were over-represented. Unsurprisingly, after weighting for non-response, the age distribution of both surveys matched the census. With respect to demographics not used in the weighting strategy, both surveys had largely consistent profiles with the Census (Tables 1 and 2), although each survey over-represented married people and, bar women in Natsal-3, under-represented single people. In each survey, retired or long-term unemployed men were under-represented whilst this group were over-represented in women. HSE 2010 over-represented those in bad or worse health.

**Table 1:** Demographic and health profile for Natsal-3, HSE 2010 and the 2011 census (where applicable): Men.

	Census 2011^a^	Before non-response weighting		After non-response weighting		
		Natsal-3	HSE 2010	Natsal-3	HSE 2010	HSE 2010
			Complete survey		Complete survey	Accepted self-completion
	%	% (95% CI)	% (95% CI)	% (95% CI)	% (95% CI)	% (95% CI)
**Demographic characteristics**						
Age group						
16–24	18.6	19.2 (18.1, 20.3)	14.5 (12.8, 16.3)	18.6 (17.5, 19.7)	18.8 (16.7, 21.1)	18.7 (16.5, 21.2)
25–34	20.9	18.7 (17.6, 19.8)	18.1 (16.5, 19.8)	20.9 (19.7, 22.2)	20.4 (18.5, 22.5)	20.1 (18.2, 22.2)
35–44	21.6	21.5 (20.1, 23.0)	23.0 (21.3, 24.7)	21.6 (20.1, 23.1)	22.0 (20.3, 23.8)	21.6 (19.8, 23.5)
45–54	21.1	21.3 (19.9, 22.8)	22.2 (20.7, 23.7)	21.1 (19.7, 22.6)	21.0 (19.5, 22.6)	21.6 (20.1, 23.3)
55–64	17.8	19.3 (18.0, 20.8)	22.3 (20.8, 23.9)	17.8 (16.5, 19.2)	17.7 (16.4, 19.2)	17.9 (16.5, 19.4)
Marital status						
Married / Civil Partnership	44.0	47.6 (46.0, 49.2)	50.6 (48.5, 52.6)	47.3 (45.7, 49.0)	47.1 (44.9, 49.3)	47.1 (44.8, 49.4)
Cohabitation	14.3	13.7 (12.6, 14.8)	15.3 (13.9, 16.8)	14.0 (12.9, 15.2)	15.6 (14.1, 17.2)	15.8 (14.2, 17.6)
Previously married / civil partner	7.6	7.8 (7.0, 8.5)	8.1 (7.1, 9.1)	7.4 (6.7, 8.1)	6.8 (6.0, 7.8)	7.2 (6.2, 8.3)
Single and never married	34.2	31.0 (29.6, 32.4)	26.1 (24.2, 28.1)	31.3 (29.9, 32.8)	30.5 (28.3, 32.9)	29.9 (27.5, 32.4)
Ethnicity						
White	85.0	86.3 (85.1, 87.5)	87.4 (85.4, 89.2)	85 (83.7, 86.3)	85.2 (82.8, 87.4)	86.5 (84.0, 88.6)
Mixed	1.8	1.7 (1.3, 2.1)	1.3 (0.9, 1.9)	1.9 (1.5, 2.3)	1.5 (1.0, 2.3)	1.5 (1.0, 2.2)
Asian/Asian British	8.5	7.6 (6.7, 8.6)	6.8 (5.4, 8.4)	8.2 (7.2, 9.3)	8.1 (6.5, 10.0)	7.4 (5.8, 9.5)
Black/Black British	3.4	3.5 (2.8, 4.2)	3.5 (2.7, 4.4)	3.9 (3.2, 4.8)	3.9 (3.0, 5.0)	3.6 (2.7, 4.7)
Other	1.3	0.9 (0.7, 1.3)	1.0 (0.6, 1.6)	1.0 (0.7, 1.4)	1.3 (0.8, 2.1)	1.1 (0.6, 1.9)
National Statistics Socio-Economic Classification						
Managerial and professional occupations	31.3	36.2 (34.6, 37.8)	36.7 (34.7, 38.7)	37.1 (35.4, 38.8)	34.7 (32.6, 36.8)	35.2 (33.1, 37.4)
Intermediate occupation	18.6	16.7 (15.4, 18.1)	17.1 (15.7, 18.6)	16.6 (15.3, 17.9)	16.8 (15.5, 18.3)	16.9 (15.4, 18.5)
Routine and manual occupations	30.5	31.9 (30.3, 33.6)	31.8 (29.8, 33.9)	31.8 (30.2, 33.5)	31.9 (29.6, 34.2)	31.6 (29.4, 33.9)
No job for last 10 years or retired	10.0	4.7 (4.1, 5.4)	5.1 (4.3, 6.1)	4.4 (3.8, 5.1)	4.5 (3.8, 5.4)	4.3 (3.6, 5.1)
Students	9.6	10.5 (9.5, 11.5)	9.3 (7.8, 11.0)	10.1 (9.2, 11.1)	12.1 (10.1, 14.4)	12.0 (9.8, 14.6)
Household tenure						
Own household	65.3	62.9 (61.2, 64.5)	67.6 (65.3, 69.8)	61.6 (59.9, 63.4)	65.9 (63.3, 68.5)	67.1 (64.4, 69.7)
Do not own	34.7	37.1 (35.5, 38.8)	32.4 (30.2, 34.7)	38.4 (36.6, 40.1)	34.1 (31.5, 36.7)	32.9 (30.3, 35.6)
Higher education level						
Degree	N/A	27.3 (25.8, 28.9)	26.2 (24.4, 28.2)	28.9 (27.4, 30.6)	26 (24.1, 28.0)	25.9 (24.0, 27.8)
Higher education, A-level/equivalent	N/A	31.0 (29.4, 32.6)	31.6 (29.6, 33.7)	30.3 (28.7, 32.0)	32.6 (30.2, 35.0)	33.6 (31.2, 36.0)
GCSE, O-level or equivalent	N/A	31.8 (30.2, 33.3)	27.3 (25.5, 29.2)	31.0 (29.5, 32.6)	27.5 (25.6, 29.4)	28.0 (26.0, 30.1)
None	N/A	9.9 (9.0, 11.0)	14.8 (13.4, 16.5)	9.7 (8.7, 10.8)	14.0 (12.4, 15.6)	12.6 (11.1, 14.3)
Household size						
1	N/A	13.8 (12.8, 14.8)	16.8 (15.1, 18.6)	13.9 (12.9, 14.9)	13.3 (11.8, 14.9)	14.0 (12.4, 15.7)
2	N/A	28.5 (27.0, 29.9)	29.6 (28.0, 31.4)	28.5 (27.0, 30.0)	27.8 (26.1, 29.7)	27.6 (25.7, 29.6)
3	N/A	21.3 (19.9, 22.7)	20.5 (18.9, 22.2)	21.4 (20.0, 22.9)	22.6 (20.6, 24.7)	22.4 (20.5, 24.5)
4	N/A	22.4 (21.0, 23.9)	21.1 (19.3, 23.0)	22.3 (20.9, 23.8)	22.5 (20.4, 24.8)	22.6 (20.4, 25.0)
5+	N/A	14.1 (12.9, 15.4)	12.0 (10.5, 13.7)	14.0 (12.7, 15.3)	13.8 (11.8, 15.9)	13.4 (11.4, 15.7)
**Health indicators**						
Self-reported general health status						
Very good/good	85.2	82.6 (81.2, 83.9)	79.4 (77.7, 81.0)	83.1 (81.8, 84.4)	81.2 (79.5, 82.7)	81.6 (79.9, 83.2)
Fair	10.4	13.7 (12.5, 15.0)	14.3 (12.9, 15.9)	13.3 (12.2, 14.6)	13.2 (11.8, 14.7)	13.5 (12.0, 15.1)
Bad or very bad health	4.4	3.7 (3.2, 4.4)	6.3 (5.4, 7.3)	3.5 (3.0, 4.1)	5.6 (4.8, 6.6)	4.9 (4.2, 5.9)
Longstanding illness	N/A	29.5 (28.0, 31.1)	36.8 (34.8, 38.8)	28.6 (27.1, 30.2)	34.5 (32.4, 36.6)	34.6 (32.5, 36.8)
Drink alcohol 3 days a week or more	N/A	27.1 (25.7, 28.6)	35.1 (33.2, 37.1)	27.0 (25.6, 28.5)	32.9 (31.0, 34.9)	33.3 (31.1, 35.5)
Smoke cigarettes nowadays	N/A	27.5 (26.0, 29.0)	24.2 (22.4, 26.0)	27.7 (26.2, 29.3)	24.6 (22.7, 26.6)	24.3 (22.3, 26.3)
Unweighted, weighted denominator		4882, 4853	2782, 2814	4882, 5746	2782, 3432	2504, 3063

All participants aged 16–64

^a^ Census participants resident in England

**Table 2:** Demographic and health profile for Natsal-3, HSE 2010 and the 2011 census (where applicable): Women.

	Census 2011^a^	Before non-response weighting		After non-response weighting		
		Natsal-3	HSE 2010	Natsal-3	HSE 2010	HSE 2010
			Complete survey		Complete survey	Accepted self-completion
	%	% (95% CI)	% (95% CI)	% (95% CI)	% (95% CI)	% (95% CI)
**Demographic characteristics**						
Age group						
16–24	18.0	16.1 (15.3 to 16.9)	13.7 (12.5 to 15.0)	17.9 (17.1 to 18.9)	17.9 (16.2 to 19.6)	17.4 (15.7 to 19.3)
25–34	20.8	19.1 (18.2 to 20.0)	19.9 (18.6 to 21.4)	20.8 (19.9 to 21.8)	20.1 (18.6 to 21.6)	19.7 (18.3 to 21.2)
35–44	21.7	23.7 (22.4 to 25.1)	22.7 (21.1 to 24.3)	21.7 (20.5 to 23.0)	22.2 (20.7 to 23.9)	22.6 (21.1 to 24.3)
45–54	21.3	23.1 (21.7 to 24.5)	24.1 (22.7 to 25.5)	21.3 (20.1 to 22.6)	21.4 (20.1 to 22.7)	21.5 (20.2 to 22.9)
55–64	18.2	18.0 (16.8 to 19.2)	19.6 (18.4 to 21.0)	18.2 (17.0 to 19.4)	18.5 (17.2 to 19.8)	18.7 (17.4 to 20.1)
Marital status						
Married / Civil Partnership	45.8	50.1 (48.6 to 51.6)	50.9 (49.1 to 52.8)	48.3 (46.8 to 49.7)	49.5 (47.6 to 51.5)	49.7 (47.7 to 51.7)
Cohabitation	14.1	12.3 (11.4 to 13.3)	14.5 (13.3 to 15.8)	12.4 (11.5 to 13.4)	15.3 (14.0 to 16.7)	15.7 (14.3 to 17.1)
Previously married / civil partner	12.4	12.5 (11.7 to 13.5)	12.7 (11.6 to 13.9)	12.1 (11.3 to 13.0)	11.0 (10.0 to 12.1)	11.3 (10.2 to 12.4)
Single and never married	27.8	25.1 (24.0 to 26.2)	21.9 (20.4 to 23.5)	27.2 (26.0 to 28.3)	24.1 (22.4 to 26.0)	23.4 (21.5 to 25.4)
Ethnicity						
White	85.1	86.4 (85.3 to 87.4)	87.7 (86.1 to 89.1)	85.1 (84.0 to 86.2)	86.6 (84.9 to 88.2)	87.8 (86.2 to 89.2)
Mixed	1.8	2.0 (1.7 to 2.4)	1.4 (1.0 to 1.8)	2.3 (1.9 to 2.7)	1.5 (1.1 to 2.0)	1.5 (1.1 to 2.0)
Asian/Asian British	8.3	6.7 (5.9 to 7.6)	6.2 (5.2 to 7.4)	7.2 (6.3 to 8.1)	6.8 (5.7 to 8.1)	6.2 (5.2 to 7.4)
Black/Black British	3.7	4.1 (3.6 to 4.7)	3.4 (2.7 to 4.2)	4.6 (4.1 to 5.2)	3.6 (2.8 to 4.5)	3.2 (2.5 to 4.2)
Other	1.0	0.8 (0.6 to 1.1)	1.3 (0.9 to 1.9)	0.8 (0.6 to 1.1)	1.5 (1.0 to 2.3)	1.3 (0.9 to 2.0)
National Statistics Socio-Economic Classification						
Managerial and professional occupations	30.6	33.5 (32.1 to 34.9)	32.4 (30.7 to 34.1)	33.2 (31.8 to 34.5)	31.2 (29.5 to 33.0)	31.7 (30.0 to 33.5)
Intermediate occupation	23.7	20.2 (19.1 to 21.3)	20.5 (19.2 to 21.8)	20.0 (18.9 to 21.1)	20.1 (18.8 to 21.5)	20.5 (19.1 to 21.9)
Routine and manual occupations	28.8	26.9 (25.6 to 28.1)	29.6 (27.9 to 31.4)	26.7 (25.5 to 27.9)	29.4 (27.6 to 31.1)	29.5 (27.7 to 31.3)
No job for last 10 years or retired	6.7	10.7 (9.8 to 11.7)	9.9 (8.8 to 11.0)	10.7 (9.9 to 11.7)	9.4 (8.4 to 10.5)	8.5 (7.6 to 9.7)
Students	10.2	8.7 (8.0 to 9.5)	7.6 (6.6 to 8.8)	9.5 (8.7 to 10.3)	9.9 (8.5 to 11.5)	9.8 (8.3 to 11.5)
Household tenure						
Own household	52.4	62.2 (60.8 to 63.6)	66.5 (64.6 to 68.4)	60.5 (59.0 to 61.9)	65.6 (63.5 to 67.7)	66.8 (64.6 to 68.9)
Do not own	47.6	37.8 (36.4 to 39.2)	33.5 (31.6 to 35.4)	39.5 (38.1 to 41.0)	34.4 (32.3 to 36.5)	33.2 (31.1 to 35.4)
Higher education level						
Degree	N/A	26.8 (25.5 to 28.0)	24.8 (23.2 to 26.5)	27.3 (26.1 to 28.6)	25.2 (23.6 to 26.8)	25.5 (23.8 to 27.2)
Higher education, A-level/equivalent	N/A	24.2 (23.0 to 25.5)	28.0 (26.3 to 29.7)	24.4 (23.1 to 25.6)	28.6 (26.9 to 30.4)	29.0 (27.2 to 30.8)
GCSE, O-level or equivalent	N/A	38 (36.6 to 39.4)	32.7 (30.8 to 34.6)	37.4 (36.1 to 38.8)	32.2 (30.4 to 34.1)	32.9 (31.0 to 34.9)
None	N/A	11 (10.2 to 11.9)	14.5 (13.2 to 15.9)	10.9 (10.0 to 11.8)	14.0 (12.7 to 15.4)	12.6 (11.4 to 14.0)
Household size						
1	N/A	10.3 (9.6 to 11.0)	10.7 (9.6 to 12.0)	10.5 (9.8 to 11.3)	9.3 (8.3 to 10.4)	9.3 (8.3 to 10.5)
2	N/A	30.3 (29.1 to 31.6)	34 (32.4 to 35.7)	30.6 (29.3 to 31.9)	33.2 (31.4 to 35.0)	33.5 (31.6 to 35.4)
3	N/A	23.1 (21.9 to 24.3)	22.5 (21.1 to 24.1)	23.1 (21.9 to 24.3)	23.2 (21.6 to 24.8)	23.0 (21.4 to 24.7)
4	N/A	23.0 (21.7 to 24.3)	20.7 (19.2 to 22.2)	22.6 (21.4 to 23.8)	21.4 (19.8 to 23.1)	21.6 (19.9 to 23.5)
5+	N/A	13.3 (12.3 to 14.4)	12.0 (10.8 to 13.4)	13.2 (12.2 to 14.3)	13.0 (11.5 to 14.7)	12.6 (11.0 to 14.3)
**Health indicators**						
Self-reported general health status						
Very good/good	84.5	82.7 (81.6 to 83.7)	78.7 (77.1 to 80.2)	83 (81.9 to 84.0)	79.4 (77.7 to 80.9)	79.6 (77.9 to 81.2)
Fair	11.1	13 (12.1 to 14.0)	15.6 (14.3 to 17.0)	12.8 (11.9 to 13.8)	15.2 (13.9 to 16.7)	15.4 (14.0 to 16.9)
Bad or very bad health	4.4	4.3 (3.7 to 4.9)	5.7 (5.0 to 6.6)	4.2 (3.7 to 4.9)	5.4 (4.7 to 6.2)	5.0 (4.3 to 5.8)
Longstanding illness	N/A	30.5 (29.2 to 31.9)	38.2 (36.5 to 39.9)	29.9 (28.6 to 31.2)	36.8 (35.0 to 38.7)	36.7 (34.8 to 38.7)
Drink alcohol 3 days a week or more	N/A	18 (16.9 to 19.2)	22.2 (20.8 to 23.7)	17.5 (16.4 to 18.7)	21.2 (19.8 to 22.6)	21.7 (20.2 to 23.2)
Smoke cigarettes nowadays	N/A	24.1 (23.0 to 25.3)	20.6 (19.2 to 22.1)	24.2 (23.1 to 25.3)	20.5 (19.1 to 22.1)	20.7 (19.1 to 22.3)
Unweighted, weighted denominator		6869, 6674	3588, 3582	6869, 5795	3588, 3418	3306, 3132

All participants aged 16–64

^a^ Census participants resident in England

Compared with Natsal-3 participants, men and women in HSE 2010 were more likely to report not having any academic qualifications or to own their home. With respect to health indicators, men and women were more likely to report bad or very bad health, and men were more likely to report a long-standing illness in HSE 2010 than in Natsal-3 (36.3%, 95% CI 34.3%-38.4%) and 30.3%, 95% CI: 28.9%-31.8%, respectively). Drinking at least three days per week was more commonly reported, and smoking cigarettes less commonly reported, by men and women in HSE 2010.

### Non-response to the self-completion module

Of all participants in HSE 2010 who were eligible for the self-completion booklet, 9% did not complete it, and therefore have no data for the sexual health questions. Participants who refused the self-completion booklet more commonly reported bad health, lower education level, and belonging to a minority ethnic group, and were less likely to own their home (S2 Table). Refusal of the self-completion booklet did not significantly change the demographic and health profile of participants (Tables 1 and 2). In contrast, 2% of eligible participants in Natsal-3 refused the CASI.

### Item non-response

Among survey participants who accepted the self-completion modules, in HSE 2010 item non-response was consistently the lowest for the contraception questions (3.6% of men and 1.6% of women) and highest for the sexual risk behaviour and STI-related indicators, ranging from 9–18% for both men and women (excluding chlamydia testing: 1.7% and 2.6% in men and women, respectively). Item non-response in Natsal-3 was typically less than 5% and consistently lower than HSE 2010, except for chlamydia testing where non-response was comparable (Table 3).

**Table 3:** Comparing item non-response for key sexual behaviours, STI-related factors and contraception use in Natsal-3 and HSE 2010, by gender.

	Men		Women	
	Natsal-3	HSE 2010	Natsal-3	HSE 2010
	% (95% CI)	% (95% CI)	% (95% CI)	% (95% CI)
**Sexual behaviour and STI related factors** ^**a**^				
Heterosexual sex before 16	1.4 (1.0, 1.9)	10.2 (9.0, 11.6)	1.6 (1.2, 2.0)	10.7 (9.5, 12.0)
Number of partners, lifetime	2.1 (1.6, 2.6)	17.6 (16.0, 19.2)	1.9 (1.6, 2.4)	14.1 (12.8, 15.6)
Number of partners, past year	1.8 (1.4, 2.4)	11.8 (10.5, 13.3)	1.9 (1.5, 2.3)	13.1 (11.8, 14.5)
Same-sex experience with genital contact, ever	0.2 (0.1, 0.5)	9.4 (8.2, 10.7)	0.3 (0.2, 0.5)	10.9 (9.8, 12.1)
Same-sex partners, past 5 years	0.3 (0.1, 0.5)	9.7 (8.6, 11.1)	0.3 (0.2, 0.5)	10.9 (9.8, 12.2)
Paid for heterosexual sex, ever	0.6 (0.4, 0.9)	13.2 (11.8, 14.9)	N/A	N/A
Paid for heterosexual sex, past 5 years	0.6 (0.4, 0.9)	13.3 (11.9, 14.9)	N/A	N/A
Ever diagnosed with a STI (excluding thrush)	1.5 (1.2, 2.0)	12.4 (11.0, 13.9)	1.4 (1.0, 1.7)	16.2 (14.9, 17.6)
Tested for chlamydia, past year^b^	0.8 (0.4, 1.4)	1.7 (1.0, 2.9)	1.0 (0.6, 1.5)	2.6 (1.9, 3.6)
**Contraception use** ^**c**^				
Usually use a contraceptive pill	1.4 (1.0, 2.0)	3.6 (2.9, 4.5)	1.3 (1.0, 1.8)	1.6 (1.1, 2.3)
Usually use male condom	1.5 (1.1, 2.1)	3.6 (2.9, 4.5)	1.4 (1.0, 1.8)	1.6 (1.1, 2.3)
Usually use female sterilisation	N/A	N/A	1.3 (0.9, 1.7)	1.6 (1.1, 2.3)
Usually use male sterilisation	N/A	N/A	1.2 (0.9, 1.7)	1.6 (1.1, 2.3)

^a^ All participants aged 16–69

^b^ Participants aged 16–44 who had one or more heterosexual partners in their lifetime

^c^ Participants who have had one or more heterosexual partner in the past year (men:16–69, women: 16–54)

N/A–Not applicable, female participants were not asked questions concerning paid sex in HSE 2010, male participants were not given the option of sterilisation in HSE 2010

**Fig 1:**
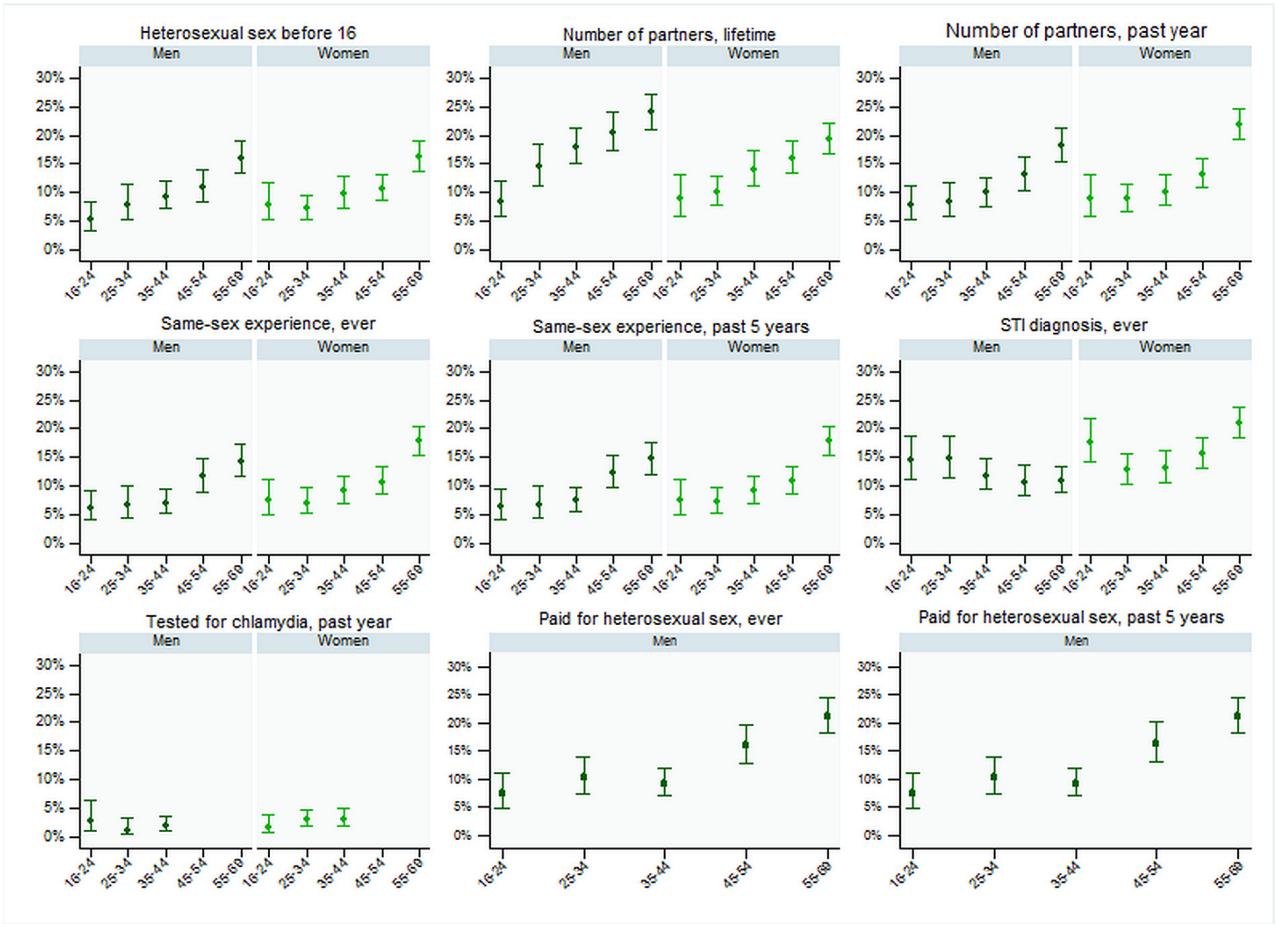
Item non-response (with 95% CI) for sexual health variables asked in HSE 2010, by gender and age-group.

Item non-response increased with age in HSE 2010 (Fig 1). For example, among those aged 16–24 years, 5.2% of men and 7.3% of women in HSE 2010 did not report their age at first sexual intercourse, but this increased to around 16% of men and women aged 55–69 years. This pattern was consistent across all sexual behaviour questions and all those relating to contraception. However, there was no difference in item non-response by age for reported STI diagnosis. Item non-response in Natsal-3 did not significantly vary with age (data not shown).

### Differences in prevalence estimates of key sexual health parameters

Median age at first sexual intercourse was broadly comparable between the two surveys: 17 years for men and 18 years for women in HSE 2010 and 17 years for men and women in Natsal-3 (Table 4). However, relative to Natsal-3, men and women in HSE 2010 were less likely to report heterosexual intercourse before 16 years (OR 0.77, 95% CI: 0.67–0.88) and (OR 0.73, 95% CI: 0.63–0.84), respectively. The reporting of high numbers of sexual partners, same-sex experience, paying for sex, STI diagnosis/es, and chlamydia testing was significantly lower in HSE 2010 than Natsal-3, although in some cases the absolute difference in prevalence estimates was small. Estimates changed little after adjustment for education and self-reported general health.

**Table 4:** Comparison of estimates of key sexual behaviours, STI-related factors, and contraception use as reported in Natsal-3 and HSE 2010, by gender.

	Men		Women	
	Natsal-3	HSE 2010	Natsal-3	HSE 2010
**Sexual behaviour and STI related factors**				
First heterosexual intercourse				
Median age at first sex (IQR)^a^	17 (16, 19)	17 (16, 20)	17 (16, 19)	18 (16, 19)
Heterosexual sex before 16				
	23.3%	18.8%	17.9%	13.7%
95% CI	21.8, 24.7	17.2, 20.6	17.0, 19.0	12.3, 15.2
OR	1.00	0.77 (0.67, 0.88)	1.00	0.73 (0.63, 0.84)
AOR	1.00	0.76 (0.66, 0.87)	1.00	0.75 (0.65, 0.86)
Unweighted, weighted denominator	5147, 6108	2479, 2954	7219, 6185	3201, 3004
Number of partners, lifetime				
0	5.9%	8.8%	4.5%	5.9%
	5.3, 6.6	7.4, 10.4	4.0, 5.0	4.8, 7.1
1	13.1%	17.2%	20.6%	23.5%
	11.9, 14.3	15.6, 18.9	19.5, 21.8	22.0, 25.2
2	8.2%	9.6%	10.7%	12.4%
	7.3, 9.1	8.3, 11.1	9.8, 11.6	11.2, 13.8
3–4	14.9%	15.8%	19.3%	22.6%
	13.7, 16.2	14.2, 17.5	18.2, 20.4	21.1, 24.2
5–9	23.0%	21.5%	24.3%	22.5%
	21.6, 24.4	19.6, 23.4	23.1, 25.4	21.0, 24.0
10+	35.0%	27.2%	20.6%	13.0%
	33.5, 36.6	25.1, 29.3	19.6, 21.7	11.7, 14.5
OR^b^	1.00	0.68 (0.61, 0.76)	1.00	0.71 (0.65, 0.78)
AOR^b^	1.00	0.68 (0.61, 0.76)	1.00	0.71 (0.64, 0.77)
Unweighted, weighted denominator	4999, 5923	2270, 2712	7041, 6021	3079, 2889
Number of partners, past year				
0	16.6%	20.2%	20.4%	21.2%
	15.5, 17.8	18.5, 22.0	19.4, 21.5	19.7, 22.8
1	67.9%	70.1%	69.9%	73.8%
	66.4, 69.3	67.9, 72.3	68.7, 71.1	72.2, 75.3
2+	15.5%	9.7%	9.7%	5.0%
	14.4, 16.6	8.2, 11.3	9.0, 10.4	4.1, 6.0
OR^b^	1.00	0.71 (0.63, 0.79)	1.00	0.81 (0.73, 0.90)
AOR^b^	1.00	0.72 (0.64, 0.81)	1.00	0.81 (0.73, 0.90)
Unweighted, weighted denominator	5020, 5939	2436, 2901	7053, 6025	3121, 2922
Same-sex experience with genital contact, ever				
%	5.6%	2.5%	6.5%	2.7%
95% CI	4.9, 6.4	1.8, 3.5	5.9, 7.1	2.1, 3.4
OR	1.00	0.44 (0.30, 0.63)	1.00	0.40 (0.31, 0.52)
AOR	1.00	0.44 (0.30, 0.64)	1.00	0.39 (0.30, 0.52)
Unweighted, weighted denominator	5192, 6172	2505, 2980	7295, 6262	3192, 2997
Same-sex partners, past 5 years				
%	2.6%	1.6%	3.5%	1.8%
95% CI	2.1, 3.1	1.0, 2.6	3.1, 4.0	1.4, 2.5
OR	1.00	0.61 (0.37, 1.03)	1.00	0.52 (0.38, 0.72)
AOR	1.00	0.63 (0.37, 1.07)	1.00	0.50 (0.36, 0.71)
Unweighted, weighted denominator	5191, 6171	2498, 2969	7293, 6261	3190, 2996
Paid for heterosexual sex, ever				
%	10.9%	5.2%	N/A	N/A
95% CI	9.9, 11.9	4.2, 6.3	-	-
OR	1.00	0.45 (0.35, 0.56)	-	-
AOR	1.00	0.45 (0.36, 0.57)	-	-
Unweighted, weighted denominator	5071, 6011	2388, 2854	-	-
Paid for heterosexual sex, past 5 years				
%	3.6%	1.9%	N/A	N/A
95% CI	3.1, 4.2	1.3, 2.6	-	-
OR	1.00	0.50 (0.35, 0.73)	-	-
AOR	1.00	0.52 (0.36, 0.76)	-	-
Unweighted, weighted denominator	5071, 6011	2386, 2851	-	-
Ever diagnosed with a STI (not including thrush)				
%	13.2%	8.0%	14.7%	11.4%
95% CI	12.1, 14.4	6.9, 9.3	13.7, 15.6	10.2, 12.8
OR	1.00	0.57 (0.47, 0.69)	1.00	0.75 (0.65, 0.87)
AOR	1.00	0.59 (0.49, 0.71)	1.00	0.78 (0.67, 0.90)
Unweighted, weighted denominator	4904, 5856	2449, 2883	6952, 5969	3006, 2819
Tested for chlamydia, past year^c^				
%	16.8%	7.1%	27.1%	15.2%
95% CI	15.4, 18.2	5.5, 9.3	25.7, 28.7	13.4, 17.3
OR	1.00	0.38 (0.28, 0.51)	1.00	0.48 (0.41, 0.57)
AOR	1.00	0.38 (0.28, 0.52)	1.00	0.46 (0.38, 0.54)
Unweighted, weighted denominator	3079, 3141	1009, 1357	4503, 3161	1464, 1470
Tested for chlamydia, past year^d^				
%	33.5%	17.2%	50.3%	26.8%
95% CI	[30.8, 36.2]	[12.9, 22.6]	[47.5, 53.1]	[22.2, 31.9]
OR	1.00	0.41 (0.29, 0.59)	1.00	0.36 (0.28, 0.47)
AOR	1.00	0.41 (0.28, 0.59)	1.00	0.34 (0.29, 0.59)
Unweighted, weighted denominator	1388, 996	323, 543	1688, 961	402, 513
**Contraception use** ^**e**^				
Usually use a contraceptive pill				
%	19.1%	17.2%	24.4%	21.2%
95% CI	17.8, 20.5	15.2, 19.5	23.1, 25.8	19.3, 23.3
OR	1.00	0.88 (0.74, 1.05)	1.00	0.83 (0.72, 0.96)
AOR	1.00	0.91 (0.76, 1.08)	1.00	0.82 (0.71, 0.95)
Unweighted, weighted denominator	3986, 4906	1882, 2232	4875, 3909	1956, 1872
Usually use male condom				
%	27.5%	26.5%	20.9%	21.5%
95% CI	25.9, 29.1	24.4, 28.7	19.6, 22.3	19.6, 23.6
OR	1.00	0.95 (0.83, 1.09)	1.00	1.03 (0.90, 1.20)
AOR	1.00	1.01 (0.88, 1.16)	1.00	1.05 (0.90, 1.21)
Unweighted, weighted denominator	3983, 4901	1882, 2232	4873, 3908	1956, 1872
Usually use female sterilisation				
%	N/A	N/A	6.0%	9.4%
95% CI	-	-	5.2, 7.0	8.1, 10.9
OR	-	-	1.00	1.63 (1.30, 2.04)
AOR	-	-	1.00	1.66 (1.32, 2.09)
Unweighted, weighted denominator	-	-	4877, 3912	1956, 1872
Usually use male sterilisation				
%	N/A	N/A	8.8%	13.0%
95% CI	-	-	7.7, 10.0	11.6, 14.5
OR	-	-	1.00	1.55 (1.29, 1.88)
AOR	-	-	1.00	1.46 (1.20, 1.78)
Unweighted, weighted denominator	-	-	4878, 3912	1956, 1872

All participants aged 16–69

^a^ Medians and quartiles calculated using survival analysis

^b^ Categorical levels modelled under the assumption of proportional odds

^c^ Participants aged 16–44 who had one or more sexual partners in their lifetime

^d^ Participants aged 16–24

^e^ Men aged 16–69 or women aged 16–54, who have had one or more heterosexual partner in the past year

N/A–Not applicable, female participants were not asked questions concerning paid sex in HSE 2010, male participants were not given the option of sterilisation in HSE 2010

AOR: adjusted for education and self-reported health

With respect to contraception method use, no differences were observed in the usual contraceptive method used reported by men, however, women were less likely to report usually using oral contraception in HSE 2010 than in Natsal-3 (OR 0.83, 95% CI: 0.72–0.96), and were more likely to report using male sterilisation and female sterilisation (Table 4). Again, adjusting for educational level and self-reported health had little effect on these results.

### Age group interaction

Differences between the two surveys in terms of the number of opposite-sex partners reported varied by age group among men. A greater proportion of men aged 16–24 years in HSE 2010 reported no sexual lifetime partners (32.9%, 95%CI: 27.2–39.1) compared with men aged 16–24 in Natsal-3 (20.3%, 95% CI: 18.0–22.7) resulting in an overall odds ratio of 0.51 (95% CI: 0.40–0.66) (S3 Table). Similar results were found in reporting no partners in the past year (OR 0.50, 95% CI: 0.39–0.99). Conversely, no differences were found between surveys in men aged 25–34 years and 35–44 years. No interactions with age were found in women.

### Comparison of participants living alone in HSE 2010 and Natsal-3

Estimates of the percentage of people living alone were similar in HSE 2010 and Natsal-3 (11.9% and 12.8%, respectively). The demographic characteristics of these participants were largely similar between surveys, although there were differences in men’s reporting of marital status, NS-SEC and smoking cigarettes, and among women, in terms of education, NS-SEC, reporting bad health and longstanding illness (S4 Table). While our findings for this subgroup analysis were similar to those presented for the whole sample in that there was generally less reporting of risky sexual behaviours in HSE 2010 than in Natsal-3, the differences between estimates for the two surveys were less pronounced in the subgroup analysis. As such, no significant differences were found between the surveys for men in lifetime partner numbers, same-sex behaviour, and paying for sex; or among women in partner numbers in the last year, same-sex partnerships in the past 5 years, and chlamydia testing in the past year (S5 Table). Contraception method use was also similar in the subgroup analysis to that for the whole sample with estimates for men similar for the two surveys, while women were less likely to report usually using the male condom and the contraceptive pill in HSE 2010, but more likely to report sterilisation.

## Discussion

These analyses have shown that it is feasible to include sexual health questions in general heath surveys such as HSE, and fairly high levels of response indicate their participants’ willingness to respond to such questions. However, relative to Natsal-3, a survey dedicated to sexual behaviour, we found greater item non-response in HSE 2010, especially among older people. We also found consistently lower reporting of risky sexual behaviours and STI-related indicators in HSE 2010 than in Natsal-3. Reporting differences may be in part due to differences in the type of people that agree to participate in these surveys as well as the different methodologies they employ.

The response rate for HSE 2010 was similar to that achieved for Natsal-3, other social surveys completed in Britain,[17,18] and the previous HSE survey in 2009,[19] suggesting little difference in overall acceptability of these surveys. Both surveys were broadly representative of the population, even though weighted to different published estimates. There were small differences in the demographic and health characteristics of those participating, including educational attainment and self-reported general health. While previous studies have shown these factors are strongly associated with sexual behaviour,[9,20] their adjustment in our analyses did not account for the differences between the surveys in prevalence estimates, suggesting that these differences are not simply due to participation bias as measured by these variables.

Nearly one in ten HSE 2010 participants refused the entire self-completion part of the survey before they knew its content, suggesting that refusal was not due to a reluctance to answer sexual behaviour questions. Furthermore, the proportion refusing the self-completion was similar to previous HSE surveys, i.e. prior to the inclusion of the sexual health module.[19,21] Of particular concern was that HSE 2010 participants who refused the self-completion module differed on some characteristics to those who completed it. To minimise bias, future HSE surveys could include further non-response weighting to adjust for the characteristics of people who refuse the self-completion module, as the HSE does when calculating estimates of gambling behaviour.[22]

Item non-response to individual questions was much higher in HSE 2010 than Natsal-3, and was associated with older age, which may introduce bias. In future, HSE could consider limiting the age-range of those completing the more sensitive sexual health questions. However, response to these questions was reasonably high even among older participants in HSE 2010, and data from older participants are vital for considering sexual health across the lifecourse.[20] With respect to the questions on contraception use, we observed high item response and more comparable reporting between surveys. Asking about contraceptive use is arguably less sensitive to reporting bias than asking about sexual behaviour, such that in Natsal these questions have (to date) been asked face-to-face.

The differences highlighted between the surveys in terms of participants’ characteristics, item non-response and reported behaviours may reflect contextual and/or methodological differences, including that Natsal is a dedicated sexual health survey, whilst HSE is a general health survey. Furthermore, potential Natsal participants are pre-notified regarding the survey’s content when invited to participate, thus those agreeing to do so may be more open to responding to questions about sexual health. This may go some way to explaining the greater item non-response in HSE, as some of these non-responders may have refused to participate in a sexual health survey if approached. Research also suggests that those who agree to take part in sexual health surveys may be more likely to engage in risky behaviour than those who refuse.[23,24] Natsal-3’s chlamydia testing estimates were higher than those observed from national surveillance data, the National Chlamydia Screening Programme (NCSP). While HSE 2010 estimates in men were similar to NCSP data, less testing was reported by women in HSE 2010 than the NCSP. (NCSP 2011/Sarah Woodhall, personal communication, 1/15/2015)

Data collection methods also differed. HSE 2010 asked its sexual health related questions in a pen-and-paper self-completion questionnaire, while Natsal-3 used CASI, bar the questions relating to first sexual intercourse and contraception. Previous studies have shown that using pen-and-paper self-completion rather than CASI elicits a greater amount of item non-response and may also result in less willingness to report socially-censured behaviours.[12,25,26] This effect may be amplified when others are present,[27] which is of relevance as within HSE 2010, all individuals in selected households were invited to participate, and concurrent interviewing encouraged, so that it is likely that the self-completion questions were answered in the presence of others. In contrast, to date, only one person is selected per household for Natsal, and interviewers encourage privacy. Using CASI in future HSEs would address this issue, but doing so is currently unfeasible as it would greatly lengthen the interview as each participant would need to use the computer in turn, resulting in some people not completing this module, and/or increasing fieldwork costs if individual computers were provided.[28] Asking participants to do the self-completion module in different rooms could be another solution, though this may not be possible in some households.

If the presence of others was the only cause of the difference in reporting, then our subgroup analysis of HSE 2010 participants who lived alone should have resulted in similar findings to those from Natsal-3. While the reduced sample size did limit the power to detect statistically significant differences within this subgroup, the differences, although smaller, were still in the direction of lower reporting in HSE 2010. Of course, living alone does not preclude others being present, and although both surveys document this, high levels of missing data prevented us from being able to account for this in our analyses. Finally, although many of the differences in the estimates between the two surveys were statistically significant, these were sometimes small in absolute terms, and thus potentially of limited importance for policy and clinical practice.

In conclusion, a number of differences exist between HSE 2010 and Natsal-3 in their context, methodology, and resulting estimates for key sexual health parameters. HSE 2010 had greater item non-response and possibly greater reporting bias than in Natsal-3, however, response in HSE 2010 was still relatively high suggesting that it is feasible to ask sexual health questions in general health surveys in England at least. Furthermore, the HSE may be reliable for measuring trends if these biases are monitored [29] and shown not to change over time. Nonetheless, methodological developments to the HSE should be considered going forward so that its data can be interpreted in conjunction with those from dedicated sexual health surveys like Natsal, thus improving our ability to monitor trends in sexual health policy and practice.[30,31]

## Supporting Information

S1 Table Question wordings, for the sexual behaviour, STI related factors and contraception use of Natsal-3 and HSE 2010. (DOCX)

S2 Table Demographic and health profile of HSE 2010 participants who accepted and refused the self-completion booklet, by gender. (DOCX)

S3 Table Reporting of numbers of partners in HSE 2010, by age group in men. (DOCX)

S4 Table Demographic and health profile of participants who lived alone in Natsal-3 and HSE-2010, by gender. (DOCX)

S5 Table Comparison of estimates of key sexual behaviour, STI-related factors, and contraceptive use as reported for participants who lived alone in Natsal-3 and HSE 2010, by gender. (DOCX)
