# Supplementary material for: Asking about Sex in General Health Surveys: Comparing the Methods and Findings of the 2010 Health Survey for England with Those of the Third National Survey of Sexual Attitudes and Lifestyles
Source: PLoS One. 2015 Aug 7;10(8):e0135203. doi: 10.1371/journal.pone.0135203 (PMC4529206; doi:10.1371/journal.pone.0135203)
Supplement: S1 Table — (DOCX) [file pone.0135203.s001.docx]

| S1 Table: Question wordings, for the sexual behaviour, STI related factors and contraception use of Natsal-3 and HSE 2010 | | | | |
| --- | --- | --- | --- | --- |
|  | Natsal-3 | | HSE 2010 | |
| Sexual behaviour and STI related factors | | |  | |
| First heterosexual intercourse | | How old were you when you first had sexual intercourse with a (woman/man)? | | How old were you when you first had sexual intercourse with someone of the opposite sex, or hasn't this happened? |
| Number of partners, lifetime | | Altogether, in your life so far, with how many (women/men) have you had sexual intercourse? | | Altogether, in your life so far, how many (women/men) have you had sexual  intercourse with (vaginal, oral or anal)? |
| Number of partners, past year | | Altogether, in the last year, with how many (women/men) have you had sexual intercourse? | | And altogether, in the last year, how many (women/men) have you had  sexual intercourse with? |
| Same-sex experience with genital contact, ever | | Have you ever had sex with a (man/women)? That is, oral or anal  sex or any other forms of genital contact. | | Have you had sex with a (man/woman) involving (genital area/penis/vaginal) contact? (That is oral (or anal) sex or any other contact involving the genital area.) |
| Same-sex partners, past 5 years | | Altogether, in the last five years, with how many men have you had sex? | | Altogether, in the last 5 YEARS, how many (men/women) have you had sex  with? |
| Paid for heterosexual sex, ever | | Have you ever paid money for sex with a woman? | | Have you ever paid money for sex with a (man/woman – opposite sex)? |
| Paid for heterosexual sex, past 5 years | | When was the last time you paid for sex with a woman? | | When was the last time you paid money for sex with a (man/woman – opposite sex)? |
| Ever diagnosed with a STI (not including thrush) | | When were you last told by a doctor or health professional that you had an infection transmitted by sex? | | Have you ever been told by a doctor or other healthcare professional that you had any of the following? |
| Tested for chlamydia, past year | | When did you have your last test for Chlamydia? | | In the last year, have you been tested for Chlamydia? |
| Contraception use | | | | |
| Usually use a contraceptive pill, male condom, female sterilisation, male sterilisation | | This is a list of possible contraception methods. Which, if any, do you (and a partner) usually use at present?   - Contraceptive pill - Male condom - Female sterilisation - Male sterilisation | | Which would you say is your most usual method these days?   - The pill - Male condom - Female sterilisation - Male sterilisation |
| For further details on question wording please see the respective publically available questionnaires | | | | |
