# Supplementary material for: Asking about Sex in General Health Surveys: Comparing the Methods and Findings of the 2010 Health Survey for England with Those of the Third National Survey of Sexual Attitudes and Lifestyles
Source: PLoS One. 2015 Aug 7;10(8):e0135203. doi: 10.1371/journal.pone.0135203 (PMC4529206; doi:10.1371/journal.pone.0135203)
Supplement: S2 Table — (DOCX) [file pone.0135203.s002.docx]

| **S2 Table:** Demographic and health profile of HSE 2010 participants who accepted and refused the self-completion booklet, by gender | | | | | | | | | | |
| --- | --- | --- | --- | --- | --- | --- | --- | --- | --- | --- |
|  | Men |  |  |  |  | Women |  |  |  |  |
|  | Accepted SC | | Refused SC | |  | Accepted SC | | Refused SC | |  |
|  | % | 95% CI | % | 95% CI | p-value | % | 95% CI | % | 95% CI | p-value |
| Age group |  |  |  |  |  |  |  |  |  |  |
| 16-24 | 17.4% | 15.3, 19.8 | 19.1% | 13.8, 25.7 | P = 0.1035 | 16.2% | 14.6, 18.0 | 20.7% | 15.4, 27.2 | P = 0.1301 |
| 25-34 | 18.8% | 16.9, 20.7 | 22.1% | 17.0, 28.2 |  | 18.3% | 17.0, 19.7 | 22.3% | 17.5, 28.1 |  |
| 35-44 | 20.1% | 18.5, 21.9 | 24.5% | 19.9, 29.8 |  | 21.1% | 19.6, 22.6 | 16.5% | 12.3, 21.8 |  |
| 45-54 | 20.1% | 18.7, 21.7 | 15.5% | 11.5, 20.5 |  | 20.0% | 18.8, 21.3 | 18.1% | 13.7, 23.6 |  |
| 55-69 | 23.6% | 22.0, 25.2 | 18.8% | 14.5, 24.0 |  | 24.3% | 22.9, 25.8 | 22.4% | 17.7, 27.9 |  |
|  |  |  |  |  |  |  |  |  |  |  |
| Marital status |  |  |  |  |  |  |  |  |  |  |
| Married / Civil Partnership | 49.0% | 46.8, 51.2 | 48.3% | 41.5, 55.2 | P = 0.0826 | 51.0% | 49.0, 53.0 | 48.5% | 42.4, 54.7 | P = 0.0328 |
| Cohabitation | 15.0% | 13.5, 16.6 | 13.1% | 9.4, 18.0 |  | 14.9% | 13.6, 16.2 | 11.1% | 7.5, 16.2 |  |
| Previously married / civil partner | 7.6% | 6.7, 8.7 | 3.8% | 2.1, 6.8 |  | 12.2% | 11.1, 13.3 | 10.5% | 7.8, 14.1 |  |
| Single and never married | 28.4% | 26.1, 30.8 | 34.7% | 27.5, 42.8 |  | 22.0% | 20.2, 23.9 | 29.8% | 24.1, 36.3 |  |
|  |  |  |  |  |  |  |  |  |  |  |
| Ethnicity |  |  |  |  |  |  |  |  |  |  |
| White | 87.2% | 84.8, 89.2 | 75.6% | 68.0, 81.9 | P = 0.0018 | 88.3% | 86.8, 89.6 | 74.7% | 66.0, 81.8 | P < 0.0001 |
| Mixed | 1.4% | 0.9, 2.1 | 2.2% | 0.9, 4.9 |  | 1.4% | 1.0, 1.9 | 1.5% | 0.4, 4.9 |  |
| Asian/Asian British | 7.1% | 5.5, 9.0 | 13.3% | 8.2, 20.7 |  | 5.9% | 5.0, 7.1 | 13.0% | 8.6, 19.1 |  |
| Black/Black British | 3.3% | 2.5, 4.4 | 6.4% | 3.5, 11.2 |  | 3.1% | 2.4, 4.1 | 7.1% | 4.3, 11.6 |  |
| Other | 1.0% | 0.6, 1.8 | 2.6% | 1.2, 5.5 |  | 1.2% | 0.8, 1.9 | 3.7% | 1.8, 7.5 |  |
|  |  |  |  |  |  |  |  |  |  |  |
| Higher education level |  |  |  |  |  |  |  |  |  |  |
| Degree | 25.3% | 23.5, 27.2 | 26.6% | 20.7, 33.4 | P < 0.0001 | 24.7% | 23.1, 26.4 | 20.7% | 15.9, 26.3 | P < 0.0001 |
| Higher education, A-level/equivalent | 32.7% | 30.5, 35.1 | 23.1% | 16.8, 30.9 |  | 27.9% | 26.3, 29.7 | 23.1% | 17.7, 29.4 |  |
| GCSE, O-level or equivalent | 27.9% | 26.0, 29.9 | 23.2% | 18.0, 29.4 |  | 32.5% | 30.7, 34.4 | 24.2% | 19.3, 30.0 |  |
| None | 14.1% | 12.5, 15.8 | 27.2% | 21.9, 33.2 |  | 14.8% | 13.5, 16.2 | 32.1% | 26.4, 38.3 |  |
|  |  |  |  |  |  |  |  |  |  |  |
| National Statistics Socio-Economic Classification | |  |  |  |  |  |  |  |  |  |
| Managerial and professional occupations | 34.8% | 32.8, 36.9 | 29.1% | 23.0, 36.1 | P = 0.4217 | 30.5% | 28.8, 32.2 | 24.4% | 19.9, 29.6 | P = 0.0001 |
| Intermediate occupation | 16.8% | 15.4, 18.3 | 16.0% | 11.8, 21.3 |  | 20.3% | 19.0, 21.7 | 16.2% | 12.5, 20.7 |  |
| Routine and manual occupations | 31.7% | 29.5, 33.9 | 34.8% | 28.8, 41.4 |  | 29.5% | 27.8, 31.2 | 28.0% | 22.9, 33.7 |  |
| No job for last 10 years or retired | 5.6% | 4.8, 6.4 | 7.7% | 4.9, 11.9 |  | 10.6% | 9.5, 11.8 | 21.2% | 16.4, 26.9 |  |
| Students | 11.2% | 9.1, 13.6 | 12.4% | 7.8, 19.1 |  | 9.1% | 7.7, 10.7 | 10.3% | 6.5, 15.8 |  |
|  |  |  |  |  |  |  |  |  |  |  |
| Household size |  |  |  |  |  |  |  |  |  |  |
| 1 | 14.3% | 12.8, 16.0 | 7.4% | 4.8, 11.1 | P = 0.0754 | 10.3% | 9.2, 11.5 | 10.1% | 7.3, 13.8 | P = 0.1121 |
| 2 | 30.7% | 28.8, 32.6 | 31.6% | 25.6, 38.3 |  | 35.8% | 34.0, 37.6 | 31.4% | 25.6, 37.7 |  |
| 3 | 21.4% | 19.5, 23.3 | 23.4% | 18.0, 29.8 |  | 21.9% | 20.4, 23.5 | 24.4% | 19.3, 30.4 |  |
| 4 | 21.1% | 19.1, 23.4 | 21.3% | 16.3, 27.4 |  | 20.3% | 18.6, 22.0 | 17.3% | 13.3, 22.3 |  |
| 5+ | 12.5% | 10.6, 14.7 | 16.3% | 11.1, 23.2 |  | 11.7% | 10.3, 13.4 | 16.8% | 12.2, 22.7 |  |
|  |  |  |  |  |  |  |  |  |  |  |
| Household tenure |  |  |  |  |  |  |  |  |  |  |
| Own household | 68.1% | 65.5, 70.6 | 56.4% | 49.7, 63.0 | P = 0.0004 | 67.9% | 65.8, 69.9 | 54.6% | 47.8, 61.2 | P = 0.0001 |
| Do not own | 31.9% | 29.4, 34.5 | 43.6% | 37.0, 50.3 |  | 32.1% | 30.1, 34.2 | 45.4% | 38.8, 52.2 |  |
|  |  |  |  |  |  |  |  |  |  |  |
| **Health indicators** |  |  |  |  |  |  |  |  |  |  |
| Self-reported general health status |  |  |  |  |  |  |  |  |  |  |
| Very good/good | 80.5% | 78.8, 82.0 | 76.9% | 71.2, 81.8 | P = 0.0005 | 79.1% | 77.4, 80.6 | 73.6% | 68.8, 77.9 | P = 0.0014 |
| Fair | 14.4% | 13.0, 15.9 | 11.3% | 8.1, 15.6 |  | 15.6% | 14.3, 17.1 | 15.9% | 12.3, 20.2 |  |
| Bad or very bad health | 5.1% | 4.4, 6.0 | 11.7% | 8.4, 16.2 |  | 5.3% | 4.6, 6.1 | 10.5% | 7.6, 14.4 |  |
|  |  |  |  |  |  |  |  |  |  |  |
| Longstanding illness | 36.5% | 34.4, 38.6 | 34.8% | 28.7, 41.4 | P = 0.6141 | 37.9% | 36.0, 39.9 | 39.7% | 34.2, 45.5 | P = 0.5591 |
| Drink alcohol 3 days a week or more | 34.4% | 32.3, 36.5 | 29.7% | 23.5, 36.7 | P = 0.2127 | 22.1% | 20.7, 23.6 | 15.5% | 11.6, 20.4 | P = 0.0134 |
| Smoke cigarettes nowadays | 23.8% | 21.9, 25.7 | 27.2% | 21.7, 33.5 | P = 0.2430 | 20.2% | 18.8, 21.8 | 19.6% | 14.9, 25.3 | P = 0.7982 |
|  |  |  |  |  |  |  |  |  |  |  |
| Unweighted, weighted denominator | 2780, 3289 | | 293, 381 | |  | 3584, 3363 | | 309, 310 | |  |
