# Supplementary material for: Asking about Sex in General Health Surveys: Comparing the Methods and Findings of the 2010 Health Survey for England with Those of the Third National Survey of Sexual Attitudes and Lifestyles
Source: PLoS One. 2015 Aug 7;10(8):e0135203. doi: 10.1371/journal.pone.0135203 (PMC4529206; doi:10.1371/journal.pone.0135203)
Supplement: S3 Table — (DOCX) [file pone.0135203.s003.docx]

| **S3 Table:** Reporting of numbers of partners in HSE 2010 and Natsal-3, by men by age group | | | | |  |  |  |  |  |  |  |  |
| --- | --- | --- | --- | --- | --- | --- | --- | --- | --- | --- | --- | --- |
|  | **Men** |  |  |  |  |  |  |  |  |  |  |  |
|  | 16-24 |  | 25-34 |  | 35-44 |  | 45-54 |  | 55-69 |  | All |  |
|  | Natsal-3 | HSE 2010 | Natsal-3 | HSE 2010 | Natsal-3 | HSE 2010 | Natsal-3 | HSE 2010 | Natsal-3 | HSE 2010 | Natsal-3 | HSE 2010 |
| **Number of partners, lifetime (%)** |  |  |  |  |  |  |  |  |  |  |  |  |
| 0 | 20.3 | 32.9 | 5.1 | 5.0 | 1.7 | 2.9 | 2.2 | 1.9 | 2.2 | 2.3 | 5.9 | 8.8 |
|  | 18.0, 22.7 | 27.2, 39.1 | 3.9, 6.6 | 2.8, 8.8 | 0.8, 3.2 | 1.4, 5.7 | 1.3, 3.8 | 1.0, 3.8 | 1.4, 3.4 | 1.4, 3.8 | 5.3, 6.6 | 7.4, 10.4 |
| 1 | 15.3 | 19.8 | 12.7 | 11.8 | 11.3 | 11.4 | 8.8 | 17.3 | 17.0 | 24.8 | 13.1 | 17.2 |
|  | 13.5, 17.2 | 15.5, 25.0 | 10.6, 15.1 | 8.6, 15.9 | 8.8, 14.5 | 8.6, 15.1 | 6.6, 11.7 | 14.0, 21.1 | 14.3, 20.1 | 21.6, 28.3 | 11.9, 14.3 | 15.6, 18.9 |
| 2 | 12.2 | 11.0 | 6.9 | 9.8 | 6.0 | 6.0 | 6.2 | 9.6 | 9.7 | 11.8 | 8.2 | 9.6 |
|  | 10.3, 14.5 | 7.5, 15.9 | 5.4, 8.7 | 7.1, 13.3 | 4.2, 8.6 | 4.1, 8.7 | 4.3, 8.7 | 7.0, 12.8 | 7.7, 12.1 | 9.5, 14.5 | 7.3, 9.1 | 8.3, 11.1 |
| 3-4 | 15.3 | 13.1 | 14.3 | 11.9 | 13.1 | 16.1 | 13.5 | 17.9 | 18.0 | 19.4 | 14.9 | 15.8 |
|  | 13.3, 17.4 | 9.5, 17.8 | 12.3, 16.6 | 8.5, 16.3 | 10.4, 16.3 | 12.8, 20.2 | 10.9, 16.6 | 14.8, 21.6 | 15.4, 21.0 | 16.4, 22.9 | 13.7, 16.2 | 14.2, 17.5 |
| 5-9 | 17.7 | 12.3 | 22.6 | 23.9 | 25.4 | 27.4 | 24.8 | 25.1 | 23.6 | 18.7 | 23.0 | 21.5 |
|  | 15.6, 20.1 | 8.7, 17.2 | 20.1, 25.2 | 19.4, 29.1 | 22.0, 29.2 | 23.2, 32.0 | 21.3, 28.7 | 21.2, 29.5 | 20.7, 26.8 | 15.8, 22.0 | 21.6, 24.4 | 19.6, 23.4 |
| 10+ | 19.2 | 10.9 | 38.4 | 37.6 | 42.5 | 36.2 | 44.5 | 28.2 | 29.4 | 23.1 | 35.0 | 27.2 |
|  | 17.1, 21.5 | 7.6, 15.3 | 35.4, 41.5 | 32.7, 42.8 | 38.5, 46.6 | 31.8, 40.8 | 40.4, 48.6 | 24.0, 32.9 | 26.2, 32.9 | 20.1, 26.3 | 33.5, 36.6 | 25.1, 29.3 |
| OR^a^ | 1.00 | 0.51 (0.40, 0.66) | 1.00 | 0.98 (0.78, 1.24) | 1.00 | 0.81 (0.64, 1.02) | 1.00 | 0.51 (0.40, 0.64) | 1.00 | 0.66 (0.55, 0.80) | 1.00 | 0.68 (0.61, 0.76) |
| AOR^a^ | 1.00 | 0.54 (0.42, 0.70) | 1.00 | 0.97 (0.76, 1.23) | 1.00 | 0.79 (0.62, 1.00) | 1.00 | 0.52 (0.41, 0.65) | 1.00 | 0.66 (0.55, 0.80) | 1.00 | 0.68 (0.61, 0.76) |
|  | 1469, 1050 | 311, 525 | 1280, 1170 | 372, 528 | 702, 1202 | 465, 543 | 647, 1166 | 468, 527 | 901, 1335 | 654, 589 | 4999, 5923 | 2270, 2712 |
|  |  |  |  |  |  |  |  |  |  |  |  |  |
| **Number of partners, past year (%)** |  |  |  |  |  |  |  |  |  |  |  |  |
| 0 | 24.6 | 39.5 | 9.7 | 9.3 | 7.4 | 8.1 | 14.6 | 14.6 | 26.5 | 30.2 | 16.6 | 20.2 |
|  | 22.2, 27.3 | 33.3, 46.0 | 8.1, 11.6 | 5.9, 14.4 | 5.6, 9.7 | 5.8, 11.3 | 12.0, 17.6 | 11.6, 18.4 | 23.6, 29.6 | 27.1, 33.5 | 15.5, 17.8 | 18.5, 22.0 |
| 1 | 42.4 | 40.6 | 71.5 | 74.9 | 81.6 | 84.6 | 74.6 | 80.9 | 66.5 | 67.2 | 67.9 | 70.1 |
|  | 39.5, 45.5 | 34.7, 46.7 | 68.6, 74.2 | 69.4, 79.6 | 78.4, 84.5 | 80.9, 87.7 | 71.0, 78.0 | 76.9, 84.4 | 63.2, 69.7 | 63.8, 70.4 | 66.4, 69.3 | 67.9, 72.3 |
| 2+ | 32.9 | 20.0 | 18.8 | 15.9 | 11.0 | 7.2 | 10.8 | 4.4 | 6.9 | 2.6 | 15.5 | 9.7 |
|  | 30.2, 35.7 | 15.4, 25.4 | 16.6, 21.2 | 12.3, 20.2 | 8.8, 13.7 | 5.2, 10.0 | 8.5, 13.6 | 3.0, 6.6 | 5.4, 8.9 | 1.6, 4.3 | 14.4, 16.6 | 8.2, 11.3 |
| OR^a^ | 1.00 | 0.50 (0.39, 0.66) | 1.00 | 0.89 (0.66, 1.20) | 1.00 | 0.75 (0.54, 1.03) | 1.00 | 0.74 (0.57, 0.98) | 1.00 | 0.75 (0.62, 0.92) | 1.00 | 0.71 (0.63, 0.79) |
| AOR^a^ | 1.00 | 0.52 (0.40, 0.68) | 1.00 | 0.89 (0.66, 1.20) | 1.00 | 0.73 (0.53, 1.01) | 1.00 | 0.73 (0.55, 0.95) | 1.00 | 0.77 (0.63, 0.94) | 1.00 | 0.72 (0.64, 0.81) |
|  | 1467, 1049 | 313, 529 | 1289, 1175 | 401, 566 | 700, 1199 | 512, 596 | 651, 1169 | 510, 576 | 913, 1347 | 700, 634 | 5020, 5939 | 2436, 2901 |
| All participants aged 16-69  ^a^ Categorical levels modelled under the assumption of proportional odds | | | | |  |  |  |  |  |  |  |  |
|  | | | | |  |  |  |  |  |  |  |  |
|  | | | |  |  |  |  |  |  |  |  |  |
