# Supplementary material for: Asking about Sex in General Health Surveys: Comparing the Methods and Findings of the 2010 Health Survey for England with Those of the Third National Survey of Sexual Attitudes and Lifestyles
Source: PLoS One. 2015 Aug 7;10(8):e0135203. doi: 10.1371/journal.pone.0135203 (PMC4529206; doi:10.1371/journal.pone.0135203)
Supplement: S4 Table — (DOCX) [file pone.0135203.s004.docx]

| **S4 Table:** Demographic and health profile of participants who lived alone in Natsal-3 and HSE 2010, by gender | | | | | | | | | | | | | | |  | | | | | | | | |
| --- | --- | --- | --- | --- | --- | --- | --- | --- | --- | --- | --- | --- | --- | --- | --- | --- | --- | --- | --- | --- | --- | --- | --- |
|  | Men |  |  | |  | Women | |  | | |  | | | |  | |  | | |  |  |  |  |
|  | Natsal-3 | HSE 2010 | P-value | |  | Natsal-3 | | HSE 2010 | | P-value | | | | |  | |  | |  |  |  |  |  |
| Age group |  |  |  | |  |  | |  | | | |  | |  |  | | | | |  |  |  |  |
| 16-24 | 8.3% | 11.8% | p = 0.177 | |  | 9.4% | | 8.0% | | P = 0.576 | | | | |  | |  | |  |  |  |  |  |
| 25-34 | 18.5% | 18.4% |  | |  | 14.6% | | 12.9% | |  | | | | |  | |  | |  |  |  |  |  |
| 35-44 | 18.4% | 20.8% |  | |  | 12.3% | | 15.4% | |  | | | | |  | |  | |  |  |  |  |  |
| 45-54 | 24.6% | 19.7% |  | |  | 18.2% | | 17.0% | |  | | | | |  | |  | |  |  |  |  |  |
| 55-69 | 30.2% | 29.4% |  | |  | 45.5% | | 46.8% | |  | | | | |  | |  | |  |  |  |  |  |
| Marital status |  |  |  | |  |  | |  | | | |  | |  |  | | | | |  |  |  |  |
| Married / Civil Partnership | 1.5% | 0.4% | p = 0.005 | |  | 1.0% | | 0.0% | | P = 0.096 | | | | |  | |  | |  |  |  |  |  |
| Cohabitation | 0.0% | 0.0% |  | |  | 0.0% | | 0.0% | |  | | | | |  | |  | |  |  |  |  |  |
| Previously married / civil partner | 41.7% | 34.7% |  | |  | 55.8% | | 53.9% | |  | | | | |  | |  | |  |  |  |  |  |
| Single and never married | 56.8% | 65.0% |  | |  | 43.2% | | 46.1% | |  | | | | |  | |  | |  |  |  |  |  |
| Ethnicity |  |  |  | |  |  | |  | | | |  | |  |  | | | | |  |  |  |  |
| White | 89.6% | 90.4% | p = 0.900 | |  | 87.0% | | 89.7% | | P = 0.463 | | | | |  | |  | |  |  |  |  |  |
| Mixed | 1.6% | 1.2% |  | |  | 2.5% | | 1.6% | |  | | | | |  | |  | |  |  |  |  |  |
| Asian/Asian British | 3.3% | 3.4% |  | |  | 3.2% | | 3.3% | |  | | | | |  | |  | |  |  |  |  |  |
| Black/Black British | 4.2% | 3.9% |  | |  | 5.3% | | 4.8% | |  | | | | |  | |  | |  |  |  |  |  |
| Chinese | 0.3% | 0.5% |  | |  | 0.9% | | 0.0% | |  | | | | |  | |  | |  |  |  |  |  |
| Other | 1.0% | 0.6% |  | |  | 1.1% | | 0.6% | |  | | | | |  | |  | |  |  |  |  |  |
| Higher education level |  |  |  | |  |  | |  | | | |  | |  |  | | | | |  |  |  |  |
| Degree | 23.7% | 25.4% | p = 0.151 | |  | 28.1% | | 24.6% | | p = 0.003 | | | | |  | |  | |  |  |  |  |  |
| Higher education, A-level/equivalent | 25.0% | 28.8% |  | |  | 19.1% | | 25.6% | |  | | | | |  | |  | |  |  |  |  |  |
| GCSE, O-level or equivalent | 32.6% | 26.4% |  | |  | 32.9% | | 25.7% | |  | | | | |  | |  | |  |  |  |  |  |
| None | 18.8% | 19.4% |  | |  | 19.9% | | 24.1% | |  | | | | |  | |  | |  |  |  |  |  |
| National Statistics Socio-Economic Classification |  |  |  | |  |  | |  | | | |  | |  |  | | | | |  |  |  |  |
| Managerial and professional occupations | 29.4% | 34.8% | p < 0.001 | |  | 32.7% | | 38.1% | | p < 0.001 | | | | |  | |  | |  |  |  |  |  |
| Intermediate occupation | 15.9% | 16.7% |  | |  | 18.1% | | 19.6% | |  | | | | |  | |  | |  |  |  |  |  |
| Routine and manual occupations | 38.4% | 39.7% |  | |  | 26.2% | | 36.4% | |  | | | | |  | |  | |  |  |  |  |  |
| Never worked and long-term unemployed | 11.6% | 0.0% |  | |  | 18.1% | | 0.0% | |  | | | | |  | |  | |  |  |  |  |  |
| Students | 4.6% | 8.8% |  | |  | 4.9% | | 5.9% | |  | | | | |  | |  | |  |  |  |  |  |
|  |  |  |  | |  |  | |  | | | |  | |  |  | | | | |  |  |  |  |
| Bad or very bad health | 9.2% | 10.7% | p = 0.346 | |  | 9.1% | | 13.0% | | P = 0.030 | | | | |  | |  | |  |  |  |  |  |
| Longstanding illness | 42.7% | 45.1% | p = 0.423 | |  | 44.5% | | 53.5% | | p = 0.004 | | | | |  | |  | |  |  |  |  |  |
| Drink alcohol 3 days a week or more | 29.3% | 33.8% | p = 0.110 | |  | 18.8% | | 19.8% | | P = 0.673 | | | | |  | |  | |  |  |  |  |  |
| Smoke cigarettes nowadays | 38.8% | 31.1% | P = 0.009 | |  | 32.5% | | 28.6% | | P = 0.126 | | | | |  | |  | |  |  |  |  |  |
|  |  |  |  | |  |  | |  | | | |  | |  |  | | | | |  |  |  |  |
| Unweighted, weighted denominator | 1108, 867 | 492, 498 |  | |  | 1238, 738 | | 444, 378 | |  | | | | |  | |  | |  |  |  |  |  |
| All participants aged 16-69 living alone | | | |  | |  |  | |  | | | |  | | |  | |  | | |  |  |  |
