# Supplementary material for: Asking about Sex in General Health Surveys: Comparing the Methods and Findings of the 2010 Health Survey for England with Those of the Third National Survey of Sexual Attitudes and Lifestyles
Source: PLoS One. 2015 Aug 7;10(8):e0135203. doi: 10.1371/journal.pone.0135203 (PMC4529206; doi:10.1371/journal.pone.0135203)
Supplement: S5 Table — (DOCX) [file pone.0135203.s005.docx]

| **S5 Table**: Comparison of estimates of key sexual behaviour, STI-related factors, and contraceptive use as reported for participants who lived alone in Natsal-3 and HSE 2010, by gender | | | | | |
| --- | --- | --- | --- | --- | --- |
|  |  |  |  |  |  |
|  | **Men** |  |  | **Women** |  |
|  | Natsal-3 | HSE 2010 |  | Natsal-3 | HSE 2010 |
| **Sexual behaviour and STI related factors** |  |  |  |  |  |
| Median age at first sex (IQR)^a^ | 17 (16, 19) | 17 (16, 20) |  | 17 (16, 19) | 18 (16, 19) |
|  |  |  |  |  |  |
| Heterosexual sex before 16 |  |  |  |  |  |
|  | 25.1% | 17.5% |  | 16.6% | 8.6% |
| 95% CI | 22.2, 28.1 | 13.8, 21.8 |  | 14.4, 19.0 | 6.1, 11.9 |
| OR | 1.00 | 0.63 (0.46 , 0.87) |  | 1.00 | 0.47 (0.32 , 0.71) |
| Unweighted, weighted denominator | 1091, 853 | 417, 429 |  | 1215, 724 | 346, 287 |
|  |  |  |  |  |  |
| Number of partners, lifetime |  |  |  |  |  |
| 0 | 5.7% | 10.8% |  | 4.4% | 4.3% |
|  | 4.3, 7.6 | 6.8, 16.8 |  | 3.3, 5.7 | 2.7, 7.0 |
| 1 | 5.6% | 7.1% |  | 11.0% | 12.9% |
|  | 4.3, 7.3 | 4.1, 11.8 |  | 9.2, 13.0 | 9.6, 17.2 |
| 2 | 6.5% | 5.2% |  | 9.7% | 14.4% |
|  | 5.1, 8.2 | 3.3, 8.2 |  | 8.0, 11.7 | 10.6, 19.3 |
| 3-4 | 12.0% | 11.3% |  | 20.4% | 25.0% |
|  | 9.9, 14.5 | 8.1, 15.5 |  | 17.9, 23.1 | 20.5, 30.1 |
| 5-9 | 22.7% | 23.5% |  | 26.3% | 23.8% |
|  | 19.9, 25.8 | 19.1, 28.7 |  | 23.7, 29.2 | 19.3, 28.9 |
| 10+ | 47.4% | 42.0% |  | 28.3% | 19.6% |
|  | 43.8, 51.0 | 36.5, 47.8 |  | 25.6, 31.1 | 15.3, 24.6 |
| OR^b^ | 1.00 | 0.77 (0.58 , 1.02) |  | 1.00 | 0.68 (0.54 , 0.85) |
| Unweighted, weighted denominator | 1018, 790 | 365, 383 |  | 1170, 691 | 330, 271 |
|  |  |  |  |  |  |
| Number of partners, past year |  |  |  |  |  |
| 0 | 34.3% | 37.7% |  | 49.6% | 51.6% |
|  | 31.0, 37.8 | 32.3, 43.3 |  | 46.5, 52.8 | 45.9, 57.2 |
| 1 | 37.2% | 42.6% |  | 36.3% | 38.1% |
|  | 34.0, 40.5 | 37.1, 48.3 |  | 33.5, 39.3 | 32.4, 44.1 |
| 2+ | 28.5% | 19.7% |  | 14.0% | 10.3% |
|  | 25.4, 31.8 | 16.0, 24.0 |  | 12.0, 16.3 | 6.9, 15.2 |
| OR^b^ | 1.00 | 0.78 (0.62 , 0.99) |  | 1.00 | 0.87 (0.68 , 1.12) |
| Unweighted, weighted denominator | 1031, 802 | 405, 419 |  | 1176, 695 | 337, 277 |
|  |  |  |  |  |  |
| Same-sex experience with genital contact, ever |  |  |  |  |  |
| % | 9.7% | 6.2% |  | 8.7% | 3.2% |
| 95% CI | 7.9, 11.9 | 4.0, 9.6 |  | 7.2, 10.5 | 1.8, 5.7 |
| OR | 1.00 | 0.61 (0.36 , 1.04) |  | 1.00 | 0.34 (0.18 , 0.65) |
| Unweighted, weighted denominator | 1103, 863 | 419, 430 |  | 1234, 735 | 349, 289 |
|  |  |  |  |  |  |
| Same-sex partners, past 5 years |  |  |  |  |  |
| % | 4.3% | 4.0% |  | 4.1% | 2.6% |
| 95% CI | 3.1, 5.8 | 2.1, 7.4 |  | 3.1, 5.6 | 1.4, 5.0 |
| OR | 1.00 | 0.93 (0.45 , 1.92) |  | 1.00 | 0.62 (0.30 , 1.29) |
| Unweighted, weighted denominator | 1102, 862 | 417, 428 |  | 1233, 734 | 349, 289 |
|  |  |  |  |  |  |
| Paid for heterosexual sex, ever |  |  |  |  |  |
| % | 17.2% | 11.8% |  | - | - |
| 95% CI | 14.7, 20.0 | 8.9, 15.6 |  | - | - |
| OR | 1.00 | 0.65 (0.45 , 0.94) |  | - | - |
| Unweighted, weighted denominator | 1051, 819 | 397, 411 |  | - | - |
|  |  |  |  |  |  |
| Paid for heterosexual sex, past 5 years |  |  |  |  |  |
| % | 7.5% | 5.0% |  | - | - |
| 95% CI | 5.7, 9.7 | 3.2, 7.8 |  | - | - |
| OR | 1.00 | 0.65 (0.38 , 1.13) |  | - | - |
| Unweighted, weighted denominator | 1051, 819 | 396, 410 |  | - | - |
|  |  |  |  |  |  |
| Ever diagnosed with a STI (excluding thrush) |  |  |  |  |  |
| % | 16.2% | 11.8% |  | 17.6% | 11.5% |
| 95% CI | 13.8, 18.9 | 8.9, 15.4 |  | 15.4, 20.1 | 8.2, 15.9 |
| OR | 1.00 | 0.69 (0.48 , 0.99) |  | 1.00 | 0.61 (0.40 , 0.91) |
| Unweighted, weighted denominator | 1011, 789 | 410, 416 |  | 1164, 688 | 340, 289 |
|  |  |  |  |  |  |
| Tested for chlamydia, past year^c^ |  |  |  |  |  |
| % | 20.2% | 10.1% |  | 35.5% | 31.2% |
| 95% CI | 16.5, 24.4 | 5.3, 18.4 |  | 30.6, 40.8 | 20.3, 44.7 |
| OR | 1.00 | 0.45 (0.21 , 0.93) |  | 1.00 | 0.82 (0.44 , 1.52) |
| Unweighted, weighted denominator | 518, 355 | 122, 142 |  | 503, 241 | 73, 71 |
|  |  |  |  |  |  |
| **Contraception use^d^** |  |  |  |  |  |
| Usually use the contraceptive pill |  |  |  |  |  |
| % | 18.7% | 20.1% |  | 24.9% | 21.8% |
| 95% CI | 15.8, 22.1 | 14.5, 27.2 |  | 21.3, 28.9 | 13.7, 32.9 |
| OR | 1.00 | 1.09 (0.70 , 1.71) |  | 1.00 | 0.84 (0.46 , 1.53) |
| Unweighted, weighted denominator | 692, 527 | 247, 253 |  | 545, 275 | 115, 104 |
|  |  |  |  |  |  |
| Usually use male condom |  |  |  |  |  |
| % | 33.0% | 35.3% |  | 29.1% | 23.7% |
| 95% CI | 29.1, 37.0 | 28.6, 42.7 |  | 24.8, 33.7 | 15.5, 34.6 |
| OR | 1.00 | 1.11 (0.78 , 1.59) |  | 1.00 | 0.76 (0.43 , 1.35) |
| Unweighted, weighted denominator | 691, 526 | 247, 253 |  | 545, 275 | 115, 104 |
|  |  |  |  |  |  |
| Usually use female sterilisation |  |  |  |  |  |
| % | - | - |  | 4.3% | 7.4% |
| 95% CI | - | - |  | 2.5, 7.1 | 3.8, 13.8 |
| OR | - | - |  | 1.00 | 1.79 (0.74 , 4.32) |
| Unweighted, weighted denominator | - | - |  | 545, 275 | 115, 104 |
|  |  |  |  |  |  |
| Usually use male sterilisation |  |  |  |  |  |
| % | - | - |  | 2.6% | 5.5% |
| 95% CI | - | - |  | 1.4, 5.0 | 2.7, 10.7 |
| OR | - | - |  | 1.00 | 2.16 (0.80 , 5.81) |
| Unweighted, weighted denominator | - | - |  | 545, 275 | 115, 104 |
| All participants aged 16-69 living alone |  |  |  |  |  |
| ^a^ Medians and quartiles calculated using survival analysis  ^b^ Categorical levels modelled under the assumption of proportional odds | | |  |  |  |
| ^c^ Participants aged 16-44 who had one or more heterosexual partners in their lifetime | | |  |  |  |
| ^d^ Men aged 16- 69 or women aged 16-54, who have had one or more heterosexual partner in the past year | | |  |  |  |
